# OSM-9 and OCR-2 TRPV channels are accessorial warm receptors in *Caenorhabditis elegans* temperature acclimatisation

**DOI:** 10.1038/s41598-020-75302-3

**Published:** 2020-10-29

**Authors:** Kohei Ohnishi, Shigeru Saito, Toru Miura, Akane Ohta, Makoto Tominaga, Takaaki Sokabe, Atsushi Kuhara

**Affiliations:** 1 Graduate School of Natural Science, Konan University, Kobe, 658-8501 Japan; 2 Faculty of Science and Engineering, Konan University, Kobe, 658-8501 Japan; 3 Institute for Integrative Neurobiology, Konan University, Kobe, 658-8501 Japan; 4 Division of Cell Signaling, National Institute for Physiological Sciences, Okazaki, Aichi 444-8787 Japan; 5 Thermal Biology Group, Exploratory Research Center on Life and Living Systems, National Institutes of Natural Sciences, Okazaki, Aichi 444-8787 Japan; 6 AMED-PRIME, Japan Agency for Medical Research and Development, Tokyo, 100-0004 Japan

**Keywords:** Genetics, Neuroscience

## Abstract

*Caenorhabditis elegans* (*C. elegans*) exhibits cold tolerance and temperature acclimatisation regulated by a small number of head sensory neurons, such as the ADL temperature-sensing neurons that express three transient receptor potential vanilloid (TRPV) channel subunits, OSM-9, OCR-2, and OCR-1. Here, we show that an OSM-9/OCR-2 regulates temperature acclimatisation and acts as an accessorial warmth-sensing receptor in ADL neurons. *Caenorhabditis elegans* TRPV channel mutants showed abnormal temperature acclimatisation. Ectopic expression of OSM-9 and OCR-2 in non-warming-responsive gustatory neurons in *C. elegans* and *Xenopus* oocytes revealed that OSM-9 and OCR-2 cooperatively responded to warming; however, neither TRPV subunit alone was responsive to warming. A warming-induced OSM-9/OCR-2-mediated current was detectable in *Xenopus* oocytes, yet ADL in *osm-9 ocr-2* double mutant responds to warming; therefore, an OSM-9/OCR-2 TRPV channel and as yet unidentified temperature receptor might coordinate transmission of temperature signalling in ADL temperature-sensing neurons. This study demonstrates direct sensation of warming by TRPV channels in *C. elegans*.

Animals sense temperature via their nervous system and other tissues to respond and adapt to ambient temperature changes. Temperature is received by a variety of temperature-sensing molecules. Transient receptor potential (TRP) channels are temperature sensors in animals that are evolutionally conserved from nematode to human^1^. The nematode *Caenorhabditis elegans* (*C. elegans*) is a good model for studying sensory mechanisms because it is amenable to powerful molecular genetic investigation. Temperature responses of *C. elegans* have been analysed with respect to various phenomena, such as thermotaxis behaviour, noxious temperature avoidance behaviour, and cold tolerance^2–6^. In thermotaxis of *C. elegans*, temperature is sensed by the AFD sensory neurons through receptor-type guanylyl cyclases (rGCs), phosphodiesterase (PDE), and cyclic-nucleotide-gated channels (CNGs), in which rGCs (*gcy-8*, *gcy-18*, and *gcy-23*) are thought to act as a temperature receptor^7^. GLR-3, a kainate-type glutamate receptor, functions as a noxious cold receptor in the ASER gustatory neurons; temperature signalling downstream of GLR-3 depends on G protein signalling, which is independent of its channel function^8^.

*C. elegans* exhibits cold tolerance and temperature acclimatisation, which are useful model for studying temperature sensation at the molecular and cellular levels^6,9^. Wild-type animals demonstrate a form of acclimatisation related to cold tolerance, which was defined as temperature acclimatisation^5,10^. For example, 15 °C-cultivated animals survive at 2 °C, whereas 25 °C-cultivated animals can not survive at 2 °C (Fig. 1a). In contrast, 15 °C-cultivated animals are transferred to 25 °C and maintained at 25 °C for 3 to 5 h, they become intolerant at 2 °C (Fig. 1a). In *C. elegans,* ASJ, ADL, and ASG sensory neurons act as temperature-responding neurons for cold tolerance and temperature acclimatisation (Fig. 1b)^5,11–14^. For example, ADL can respond to temperature stimuli, as indicated by an increased Ca^2+^ concentration in ADL upon warming^12^, which leads to changes of cold tolerance and temperature acclimatisation.

**Figure 1:**
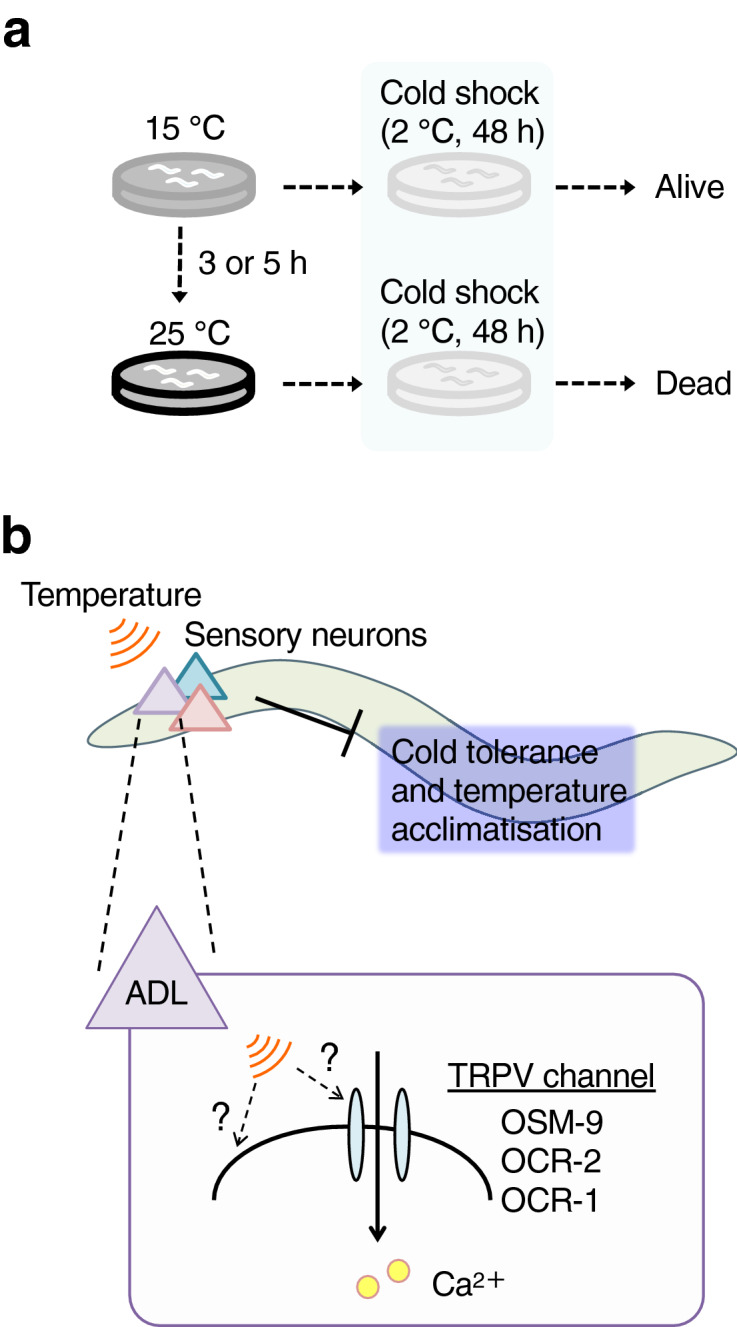
Thermosensory neurons regulate cold tolerance and temperature acclimatisation in *C. elegans*. (**a**) Cultivation conditions for the temperature acclimatisation assay. 15 °C-cultivated N2 wild-type animals can survive at 2 °C. 25 °C-cultivated N2 wild-type animals failed to survive at 2 °C. When 15 °C-cultivated wild-type animals were transferred and conditioned at 25 °C for 3 or 5 h, they exhibited decreased survival at 2 °C. (**b**) Cold tolerance and temperature acclimatisation are regulated by a subset of sensory neurons. ADL sensory neurons can detect temperature, and TRPV channels in ADL are involved in temperature acclimatisation.

TRPV channels OSM-9, OCR-1, and OCR-2 act in temperature sensation by ADL (Fig. 1b)^12,15^. Thermal-dependent Ca^2+^ concentration changes in ADL upon warming stimuli in *osm-9 ocr-2; ocr-1* triple mutant animals were decreased compared with wild-type ADL^12^. Cultivation of OSM-9/OCR-1/OCR-2 mutants at 25 °C resulted in abnormal temperature acclimatisation and decreased ADL neural activity in response to temperature stimuli, suggesting that TRP channels are necessary for ADL temperature signalling for temperature acclimatisation^12,15^. However, it remains unclear whether these TRPs act as temperature receptors in ADL.

OSM-9 and OCR-2 belong to the TRPV subfamily, which contains multiple heat sensors in mammals; TRPV4, the closest homologue of OSM-9, is involved in thermosensation and mechanosensation^16–22^. OSM-9 is expressed in many sensory neurons involved in olfaction, osmosensation, and mechanosensation^21^. OSM-9/capsaicin receptor-related genes *ocr-1*, *ocr-2*, *ocr-3*, and *ocr-4* are largely co-expressed with *osm-9*. OSM-9 and OCR-2 are thought to cooperatively function in the ASH nociceptive neurons and AWA chemosensory neurons^23^. In ASH and ADL sensory neurons, OSM-9 and OCR-2 are required to sense food shortages and population density increases^24^. In FLP and PHC neurons, OSM-9 and OCR-2 also mediate thermal avoidance behaviour^25^. However, electrophysiological analysis of *Xenopus* oocytes and HEK293 cells has not resulted in detectable currents through OSM-9 and/or OCR-2 upon stimulation with heat, voltage, thapsigargin, IP3, capsaicin, or high osmotic strength solutions^21,23,26^.

We show here that the OSM-9/OCR-2 TRPV channels act as a temperature receptors and are involved in temperature acclimatisation of *C. elegans*. Ectopic expression of OSM-9 and OCR-2 confers a temperature response in non-warmth-sensing neuron. Electrophysiological studies employing a *Xenopus* oocyte expression system demonstrated that OSM-9/OCR-2 TRPV responds to warming. These findings demonstrate direct warm sensation by TRPV channels in *C. elegans*, which negatively controls individual temperature acclimatisation.

## Results

### Temperature acclimatisation of TRPV channel mutants at 15 °C

*C. elegans* exhibits cold tolerance and temperature acclimatisation (Fig. 1a). Wild-type animal survives at 2 °C after cultivation at 15 °C, while they can not survive at 2 °C after cultivation at 25 °C (Fig. 1a). Besides, 15 °C-cultivated wild-type animals are transferred to 25 °C and stayed at 25 °C for 3 to 5 h, they become intolerant at 2 °C (Fig. 1a). 25 °C-cultivated *osm-9* mutants exhibit abnormal enhancement of cold tolerance, suggesting that OSM-9 activation inhibits cold tolerance after cultivation at a warm temperature, as previously reported^12^. However, *osm-9* mutants exhibit normal cold tolerance after cultivation at 15 °C, a lower temperature^5^.

**Figure 2:**
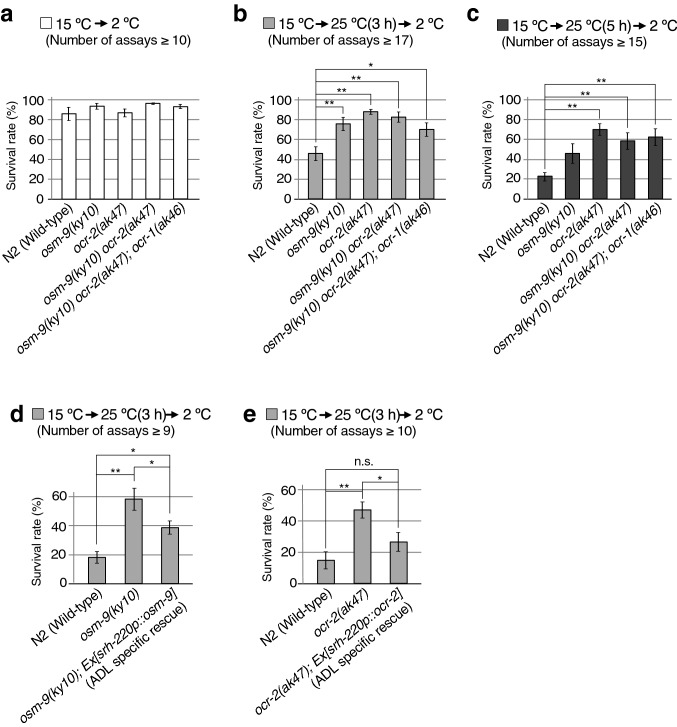
Temperature acclimatisation assay. Animals were assayed using the 15 °C → 25 °C (0, 3, or 5 h) → 2 °C protocols. (**a**,**b**,**c**) TRPV mutants *osm-9(ky10)*, *ocr-2(ak47), osm-9(ky10) ocr-2(ak47)* and *osm-9(ky10) ocr-2(ak47); ocr-1(ak46)* showed abnormally elevated survival rate (Number of assays ≥ 9, mean ± SEM). Comparisons were performed with Dunnett’s test for each condition: 15 °C → 25 °C (0, 3, 5 h) → 2 °C (**p < 0.01). (**d**,**e**) The abnormally elevated cold tolerance of *osm-9* and *ocr-2* mutants was partially rescued by expression of *osm-9* and *ocr-2* cDNA in ADL sensory neurons, respectively (number of assays ≥ 9, mean ± SEM). Statistical significance was assessed using ANOVA followed by a Bonferroni multi-comparison test (n.s. *p* ≥ 0.05, **p < 0.01, *p < 0.05).

To observe a strong phenotype in *osm-9* mutants, we performed a temperature acclimatisation test (Fig. 2). Wild-type animals grown at 15 °C were transferred and maintained at 25 °C for 0, 3, or 5 h and then exposed to a cold shock of 2 °C for 48 h [15 °C → 25 °C(0, 3, or 5 h) → 2 °C]. The survival rates of wild-type animals conditioned at [15 °C → 2 °C], [15 °C → 25 °C(3 h) → 2 °C], or [15 °C → 25 °C(5 h) → 2 °C] were approximately 86%, 46%, and 23%, respectively (Fig. 2a–c)^5,10,15^. In contrast, *osm-9* mutant animals exhibited abnormally elevated cold tolerance under the 15 °C → 25 °C(3 or 5 h) → 2 °C protocol (Fig. 2b,c). Similarly, the cold tolerance of *ocr-2* mutants, defective for another ADL TRPV, was elevated under the same protocols (Fig. 2b,c). We also found that the *osm-9 ocr-2* double mutant and *osm-9 ocr-2; ocr-1* triple mutant showed almost similar phenotype as single mutants (Fig. 2b,c), indicating that *osm-9* and *ocr-1, 2* function together in a genetic pathway.

The abnormally elevated cold tolerance of *osm-9* and *ocr-2* mutants was partially rescued by expression of *osm-9* and *ocr-2* cDNA in ADL, respectively (Fig. 2d,e). These results suggest that OSM-9 and OCR-2 function in ADL during normal cold acclimatisation of wild-type animals, and imply that expression of OSM-9 and OCR-2 in neurons other than ADL is also required. Alternatively, it is possible that expression from the transgene is either too high or too low for full rescue.

### Thermosensitivity of ADL sensory neurons in TRPV mutants

As previously reported in Fig. 2D of Ujisawa et al.^12^, thermal-dependent Ca^2+^ concentration changes in ADL upon a 6 °C range-warming stimuli in the *osm-9 ocr-2; ocr-1* triple mutant revealed decreased thermal responses compared with wild-type^12^. Additionally, Okahata et al. Figure S6^15^ described that *osm-9* and *ocr-2* single mutants and the *osm-9 ocr-2* double mutant exhibited normal phenotypes with regard to ADL thermal responses upon the same 6 °C range-warming^15^.

**Figure 3:**
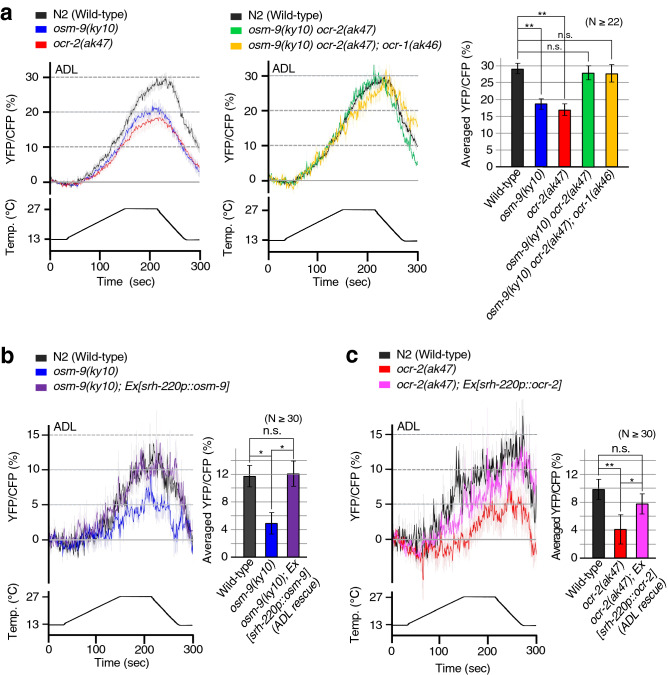
Ca^2+^ imaging of ADL sensory neurons in TRPV mutants. Average thermal responses in ADL of each strain cultivated at 15 °C. Line graphs indicate YFP/CFP ratio changes under warming and cooling. (**a**) Data for wild-type in left and middle graphs are the same, given that the experiments were conducted simultaneously. Comparisons were performed with Dunnett’s test (n.s. *p* ≥ 0.05, **p < 0.01). The bar graph indicates the average ratio change from 230 to 235 s, a maximum point of ratio changes in wild-type (n ≥ 22, mean ± SEM). (**b**,** c**) Abnormal temperature responses of ADL in *osm-9* and *ocr-2* mutants were rescued by expression of *osm-9* and *ocr-2* cDNA in ADL, respectively. The bar graph indicates the average ratio change from 230 to 235 s (n ≥ 30, mean ± SEM). Statistical significance was assessed using ANOVA followed by a Bonferroni multi-comparison test (n.s. *p* ≥ 0.05, **p < 0.01, *p < 0.05).

We used a wide range of warming stimuli (an approximately 14 °C range) for Ca^2+^ imaging (Fig. 3) to detect any abnormalities in *osm-9* and *ocr-2* single mutants. We monitored thermal responses of ADL in single *osm-9* and *ocr-2* mutant animals using a genetically encoded Ca^2+^ indicator, YC3.60. As a result, *osm-9* and *ocr-2* single mutants exhibited decreased thermal responses compared with wild-type animals under a wide range of warming stimuli from 13 to 27 °C (Fig. 3a).

Abnormal thermal responses of ADL in *osm-9* and *ocr-2* mutants were rescued by expression of *osm-9* and *ocr-2* cDNA in ADL, respectively (Fig. 3b,c), suggesting that OSM-9 and OCR-2 function in temperature signalling of ADL. However, we unexpectedly found that the *osm-9 ocr-2* double mutant and *osm-9 ocr-2; ocr-1* triple mutant showed normal ADL thermal responses under identical warming stimuli (Fig. 3a). It is possible that compensatory mechanisms lead to expression of other TRPV subunits, which induce temperature-dependent changes in Ca^2+^ concentration in ADL. Alternatively, it is possible that a lack of TRP signalling is compensated by an unidentified other temperature sensing mechanism, as OCR-1, OCR-2, and OSM-9 are the only TRPV channels expressed in ADL. Although TRP triple mutant showed thermal response of ADL at a level in optical Ca^2+^ imaging using yellow cameleon YC3.60, detailed-electrophysiological feature of ADL still could not be completely restored in TRP triple mutant, which could cause their abnormally elevated cold tolerance of TRP triple mutant (Fig. 2b,c).

### Expression of OSM-9 and OCR-2 is sufficient to confer temperature responsiveness to non-temperature sensing neurons

To investigate whether TRPVs are capable of conferring thermal sensitivity to warm stimuli, we expressed OSM-9 and OCR-2 TRPVs in the right ASE (ASER) gustatory neuron. ASER was used because it is a non-warmth-sensing neuron that has previously been used in reconstitution analysis to measure temperature sensitivities of novel temperature receptors, such as rGCs and a degenerin/epithelial Na^+^ channel-type mechanoreceptor involved in thermotaxis and cold tolerance, respectively^7,13^. ASER acts as a cool-sensing neuron in which GLR-3, a kainate-type glutamate receptor, functions as a cool-sensing receptor^8^. We used a *glr-3* mutant in which ASER becomes a non-thermosensitive neuron due to loss of its cold receptor.

**Figure 4:**
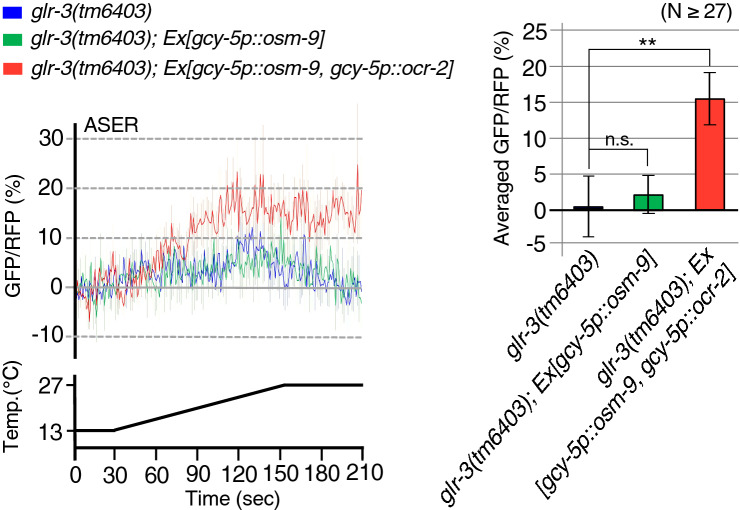
Ca^2+^ imaging of a gustatory neuron additively expressing OSM-9 and OCR-2. *osm-9* cDNA or *osm-9* cDNA with *ocr-2* cDNA were expressed in a non-warmth-sensing gustatory neuron, ASER of a *glr-3* mutant lacking a cold receptor GLR-3. Ca^2+^ imaging was performed using G-CaMP8. Line graphs indicate the G-CaMP8/tagRFP ratio change under warming. The bar graph indicates the average ratio change from 181 to 200 s, a temperature maximum point (n ≥ 27, mean ± SEM). Comparisons were performed with Dunnett’s test (n.s. *p* ≥ 0.05, **p < 0.01).

**Figure 5:**
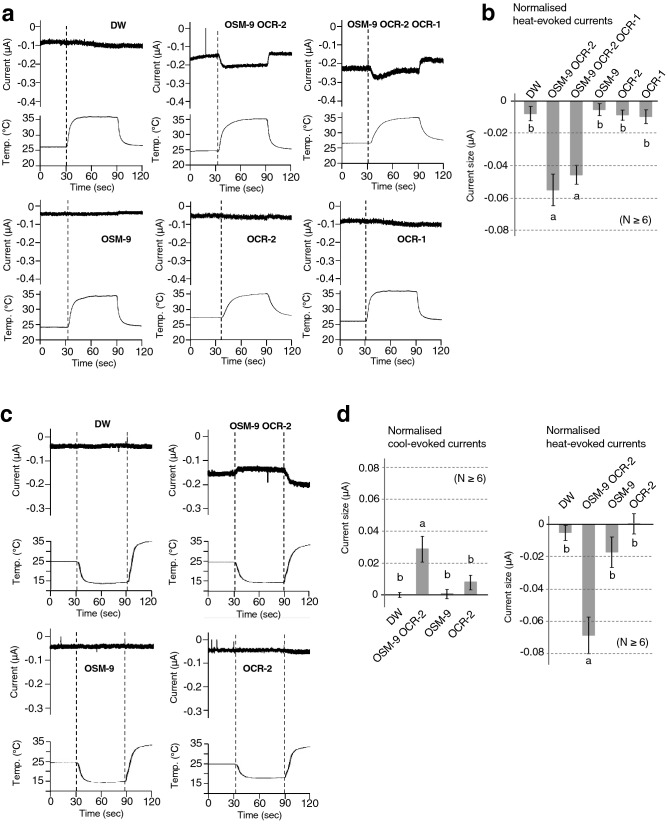

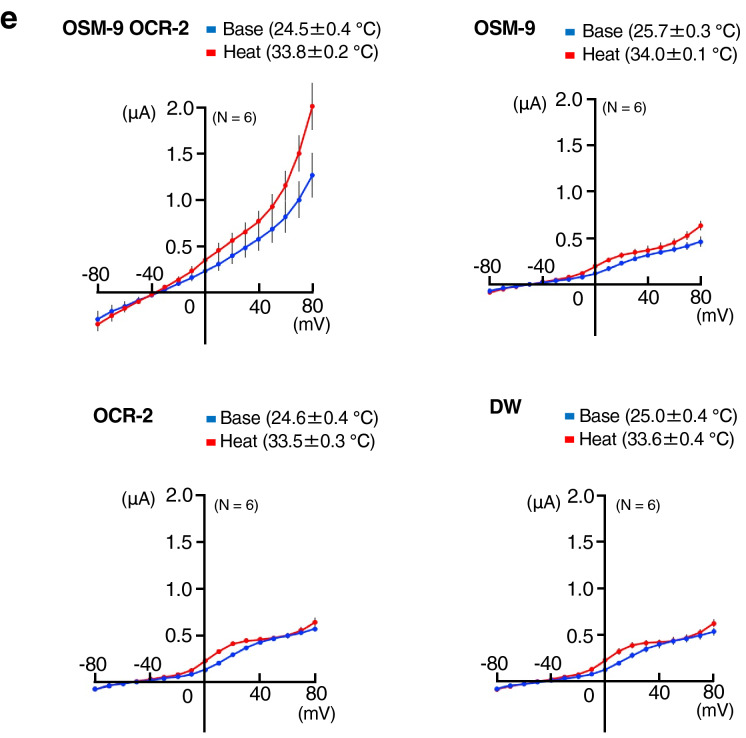
Electrophysiological analysis of TRPV OSM-9, OCR-2, and OCR-1 using *Xenopus* oocytes. (**a**) Representative traces of currents (upper) and temperature (lower) for distilled water (DW)-injected *Xenopus* oocytes or *Xenopus* oocytes expressing OSM-9, OCR-2, OCR-1, OSM-9/OCR-2, or OSM-9/OCR-2/OCR-1. The membrane potential was set at − 60 mV. (**b**) Comparison of normalised warming-evoked currents in DW-injected *Xenopus* oocytes and *Xenopus* oocytes expressing OSM-9, OCR-2, OCR-1, OSM-9/OCR-2, or OSM-9/OCR-2/OCR-1 (n ≥ 6 oocytes per group, mean ± SEM). Amplitudes of warming-evoked currents were calculated by subtracting the peak inward currents at basal temperature (approximately 25 °C) from the peak inward currents after temperature changes for each *Xenopus* oocyte. Statistical significance was assessed using ANOVA followed by a Bonferroni multi-comparison test for results detected between groups marked with “a” and “b” (*p* < 0.05). (**c**) Representative traces for cool- or warm-stimulation (upper) and temperature (lower) for DW-injected *Xenopus* oocytes or *Xenopus* oocytes expressing OSM-9, OCR-2, or OSM-9/OCR-2. The membrane potential was set at − 60 mV. (**d**) Comparison of normalised cool-evoked currents in DW-injected *Xenopus* oocytes and *Xenopus* oocytes expressing OSM-9, OCR-2, or OSM-9/OCR-2 (n ≥ 6 oocytes per group, mean ± SEM). Amplitudes of cool-evoked currents were calculated by subtracting the peak inward currents at basal temperature (approximately 25 °C) from the peak inward currents at approximately 15 °C for each *Xenopus* oocyte (left panel). Amplitudes of warming-evoked currents were calculated by subtracting the peak inward currents at approximately 15 °C from the peak inward currents at approximately 35 °C for each *Xenopus* oocyte (right panel). Statistical significance was assessed using ANOVA followed by a Bonferroni multi-comparison test for results detected between groups marked with “a” and “b” (*p* < 0.01). (**e**) Averaged current–voltage (I–V) relationships for DW-injected *Xenopus* oocytes or for *Xenopus* oocytes expressing OSM-9, OCR-2, or OSM-9/OCR-2 in response to warm stimuli. Ramp pulses from − 80 to + 80 mV were applied at 3-s intervals and I-V curves were obtained at indicated temperatures. The blue traces represent the I–V relationship at room temperature, while the red traces represent the warming-evoked I–V relationship (n ≥ 6 oocytes per group, mean ± SEM).

To detect Ca^2+^ levels in the ASER neurons of *glr-3* mutants, we expressed OSM-9 and OCR-2 with G-CaMP8 using an ASER-specific promoter. As endogenous OSM-9 is expressed in ASER of wild-type animals, we confirmed whether excess expression of the *osm-9* gene in ASER of *glr-3* mutants conferred warmth sensitivity to ASER. The *glr-3* mutants overexpressing OSM-9 in ASER did not respond to warming stimuli (Fig. 4), similar to ASER in *glr-3* mutants, which served as a negative control. This result suggests that expression of only OSM-9 in ASER is not enough to confer responsiveness to warming. However, we found that ASER neurons in animals expressing *ocr-2* in addition to *osm-9* were responsive to warming stimuli (Fig. 4). Therefore, we concluded that expression of OSM-9 and OCR-2 TRPV channels is sufficient to confer temperature responsiveness to non-thermally sensitive neuron. We next employed electrophysiological analysis of *Xenopus* oocytes to investigate whether OSM-9 and OCR-2 cooperatively act as a channel for temperature sensing (Fig. 5).

### Thermal stimuli evoked inward currents in *Xenopus* oocytes co-expressing OSM-9 and OCR-2

We conducted electrophysiological analysis to evaluate the thermosensitivity of OSM-9 and OCR-2 by employing two-electrode voltage clamp recording of *Xenopus* oocytes (Fig. 5a–e). Previous electrophysiological studies have not detected currents via OSM-9 and/or OCR-2 upon stimulation with heat^21,23,26^. Our analysis also demonstrated that a warm stimulus (~ 36 °C) did not evoke any detectable inward current in *Xenopus* oocytes separately injected with *osm-9* or *ocr-2* cRNA alone, similar to *Xenopus* oocytes injected with distilled water (DW) as a control (Fig. 5a,b). In contrast, *Xenopus* oocytes simultaneously injected with both *osm-9* and *ocr-2* cRNA exhibited inward currents upon warm stimulation up to approximately 35 °C; inward currents arose just after the onset of thermal stimulation (Fig. 5a, OSM-9 OCR-2). A previous report showed that OSM-9, OCR-1, and OCR-2 cooperatively control thermosensory activity in *C. elegans*, with OCR-1 acting as a negative regulator of TRPV channels^13,16^, although it remains unclear whether OCR-1 forms a heterochannel complex with OSM-9 and OCR-2. A warm stimulus (~ 36 °C) evoked detectable inward currents in *Xenopus* oocytes simultaneously injected with *osm-9*, *ocr-2,* and *ocr-1* cRNA (Fig. 5a,b, OSM-9 OCR-2 OCR-1), similar to *Xenopus* oocytes injected with both *osm-9* and *ocr-2* cRNA (Fig. 5a,b, OSM-9 OCR-2), indicating that no significant effect was detected with OCR-1.

Notably, *Xenopus* oocytes co-expressing OSM-9 and OCR-2 showed basal currents at room temperature (approximately 25 °C), which was not observed in *Xenopus* oocytes injected with *osm-9* or *ocr-2* cRNA alone (Fig. 5a). These results raised the possibility that the thermal activation threshold of these two channels is much lower than room temperature (approximately 25 °C). Therefore, we introduced a cooling stimulus before warm stimulation to the *Xenopus* oocytes expressing OSM-9 and OCR-2. We found that *Xenopus* oocytes simultaneously injected with both *osm-9* and *ocr-2* cRNA evoked inward currents in response to a warm stimulus (approximately 36 °C) after a cooling stimulus (approximately 15 °C) (Fig. 5c OSM-9 OCR-2, d right panel). Again, inward currents were elicited just after the onset of warm stimulation, indicating that these two channels did not possess apparent thermal thresholds for activation. In *Xenopus* oocyte injected with both *osm-9* and *ocr-2* cRNA, a slight decrease in inward currents was observed upon cooling stimulation, suggesting one possibility that the temperature threshold for activation is lower than 15 °C (Fig. 5c,d left panel).

To evaluate the current–voltage (I–V) relationship to warm stimuli, we applied ramp pulses from − 80 to + 80 mV during 0.5-s at 3-s intervals. The I–V relationship of OSM-9- and OCR-2-injected *Xenopus* oocytes showed outward rectification at the basal temperature (24.5 ± 0.4 °C; Fig. 5e, blue trace) that was augmented by warm stimulation (33.8 ± 0.2 °C; Fig. 5e, red trace) compared with the basal I–V relationship (Fig. 5e, blue trace). DW-, OSM-9-, or OCR-2-injected *Xenopus* oocytes did not show such clear outward rectification at either basal or experimental temperatures (Fig. 5e, blue and red traces).

## Discussion

Our findings indicate that OSM-9 and OCR-2 TRPV channels cooperatively function as a temperature receptor. Electrophysiological analysis of *Xenopus* oocytes indicated that OSM-9/OCR-2 TRPV channels have an ability to react to temperature stimulation. A loss of either OSM-9 or OCR-2 induced abnormal thermosensation in the ADL sensory neuron, which causes a resulting disruption of acclimatisation. These results demonstrate that TRPV channels in *C. elegans* can be directly activated by warm stimuli, which correlates with temperature responsiveness at the animal level.

Homo- or hetero-multimerisation and complex assembly have been confirmed for many TRP channels in various species. The first to be identified was an eye-specific TRP and TRPL in *Drosophila*^27^; specifically, a combination of TRP homomultimers and TRP-TRPL heteromultimers produce light-induced currents. TRPV subfamily members, such as human TRPV5 and TRPV6, undergo homo- or hetero-complex assembly^28–31^. Previous reports and the results of this study indicate that OSM-9 and OCR-2 form heteromultimers, or each channel forms homomultimers that function cooperatively with one another. In *C. elegans*, both OSM-9 and OCR-2 are required for chemosensation in AWA sensory neurons, as well as mechanosensation and osmosensation in ASH sensory neurons. Ciliary colocalisation of OSM-9 and OCR-2 is codependent^23^, suggesting that OSM-9 and OCR-2 form heteromeric complexes. Another *C. elegans* heteromeric TRPV channel, consisting of OSM-9 and OCR-4, was shown to be a receptor for nicotinamide (NAM, a form of vitamin B3 and an endogenous metabolite) in a heterologous *Xenopus* oocyte system. OSM-9/OCR-4 regulates NAM-induced cell death in uterine vulval one (uv1) and OLQ neurons in *C. elegans*^32^. However, *Xenopus* oocytes expressing OSM-9 or OCR-4 did not respond to NAM. Stoichiometry of these channels inferred using total internal reflection (TIRF) microscopy with GFP-labelled OSM-9 and OCR-4 demonstrated that OSM-9 and OCR-4 channels may function with two subunits of each in the active channel^32^. These previous reports are consistent with the results of this study, which show that OSM-9 and OCR-2 channels can together respond to heat, but that each channel on its own cannot. The warming-evoked current–voltage relationship obtained from *Xenopus* oocytes co-expressing OSM-9 and OCR-2 showed an outward rectification that is typical to vertebrate TRPV channels^16,17,33^, suggesting that OSM-9/OCR-2 form a warmth-sensitive TRP channel.

Warming-evoked currents arose just after temperature elevation from room temperature or cooling stimulus in *Xenopus* oocytes expressing OSM-9/OCR-2 (Fig. 5a,c, OSM-9 OCR-2). This raises two possibilities: the temperature threshold for activation is lower than 15 °C, or OSM-9/OCR-2 might not have a fixed temperature threshold for activation and can react to warming at any temperature. The former possibility matches well with the fact that *Xenopus* oocytes simultaneously injected with *osm-9* and *ocr-2* showed outward rectifying currents at 25 °C without warm stimulation (Fig. 5e, OSM-9 OCR-2), indicating that OSM-9/OCR-2 is at least partially activated at this temperature. The immediate response from 25 to 15 °C (Fig. 5c, OSM-9 OCR-2) could also support this idea that the temperature threshold for activation is lower than 15 °C. In this case, OSM-9/OCR-2 sensitivity is very different to the living temperature of *C. elegans* from 15 to 25 °C. This discrepant sensitivity may have been caused by the difference in membrane lipid composition between *C. elegans* and *Xenopus* oocyte or the intracellular/extracellular condition in electrophysiological measurement. There is, however, a technical obstacle to test this possibility as temperature lower than 15 °C often evokes endogenous responses in *Xenopus* oocytes.

The later possibility, OSM-9/OCR-2 might not have a fixed temperature threshold for activation and can react to warming at any temperature, is supported by the fact that the current size evoked by warming from 15 °C was comparable to that evoked from 25 °C (Fig. 5d, right panel). In this case, OSM-9/OCR-2 may be constitutively active channel which resulted in a relatively large leak currents in *Xenopus* oocytes expressing these two channels, similar to a phenomenon described for vertebrate TRPVs^34^. This can explain why leak currents were still larger in OSM-9/OCR-2 injected *Xenopus* oocytes compared to OSM-9- or OCR-2-injected oocytes even under low temperature condition (Fig. 5c, OSM-9 OCR-2).

Although the OSM-9/OCR-2 channel was responsive to thermal stimuli, its current size was small. The functional expression level of OSM-9 and OCR-2 might simply be inefficient. Alternatively, activity of the OSM-9/OCR-2 channel could be enhanced by unidentified upstream molecules that also sense temperature in vivo; Many TRP channels are regulated by upstream GPCR and G protein-coupled signalling via second messengers. In *Drosophila* phototransduction, a GPCR (rhodopsin) and its downstream trimeric G protein signalling regulate the gating of TRP and TRPL channels. Opening of the TRP channels depends on Gq and phospholipase C (PLC) to produce a light-induced current^35–39^. Recent reports have claimed that a thermotactic behaviour in *Drosophila* larva to move towards an optimal temperature relies on a signalling cascade that includes rhodopsin, Gq, PLC, and the TRPA1 channel^40,41,42^. In mammals, GPCR-TRP sensory signalling for detecting noxious, irritant, and inflammatory stimuli in the skin, gastrointestinal, and respiratory systems have been reviewed^43^. Many types of GPCRs expressed in nociceptive neurons are activated by noxious stimuli, such as proteases, peptides, purines, and lipids^44,45^. These GPCR signalling amplify or sensitise downstream components including TRP channels, which amplify or maintain GPCR signalling. For instance, cAMP-dependent protein kinase A (PKA) or PKC phosphorylate TRP channels to reduce their activation threshold in response to endogenous agonists^44^.

Previous experiments in *C. elegans* indicate that TRPVs may act downstream of G protein signalling; indeed, G protein-coupled receptor kinase 2 (GRK-2) and regulator of G protein signalling 3 (RGS-3) were shown to directly or indirectly modulate TRPV channel activity^46,47^. In the AWA chemosensory neurons of *C. elegans*, chemical cues are likely to be received by GPCRs, whose signals are transmitted to downstream trimeric G proteins ODR-3 and GPA-3, which then open OSM-9/OCR-2 channels^48,49^. Moreover, the G-protein α subunit GOA-1 in the ASH nociceptive neurons plays a role in avoidance behaviour of *C. elegans* against strong alkaline pH, and may function upstream of OSM-9/OCR-2^50^. In these cases, GPCRs and G protein-coupled signalling are thought to function upstream of TRP channels.

**Figure 6:**
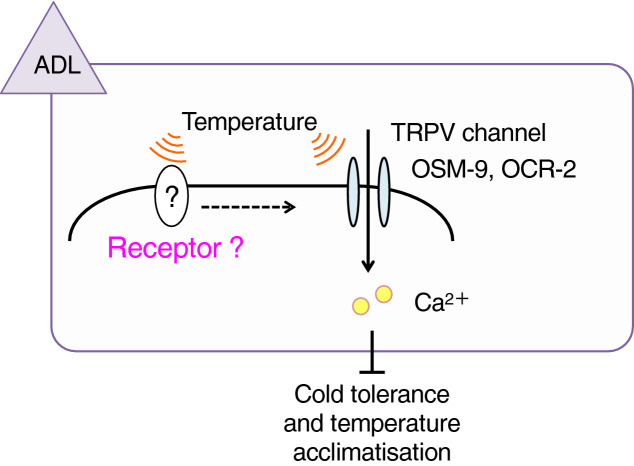
Model of temperature sensation in ADL neurons for cold tolerance and temperature acclimatisation modulated by TRPV channels and unidentified temperature receptors, such as GPCRs. Temperature is sensed by both unidentified GPCRs and OSM-9/OCR-2 TRPV channels. GPCR-mediated G protein signalling regulates OSM-9/OCR-2 activity, which controls cold tolerance and temperature acclimatisation.

With regard to the cold tolerance of *C. elegans*, a *gpa-3* mutant, which lacks a trimeric G protein α subunit, showed abnormal cold tolerance that was partially rescued by expressing a *gpa-3* cDNA in the ASJ thermosensory neurons^11^. We speculate that an unidentified temperature receptor, such as a GPCR, acts upstream of GPA-3 in ASJ. GPA-3 is also expressed in the ADL thermosensory neurons; therefore, it is possible that GPA-3 associates with the temperature signalling pathway in ADL. If there is an unidentified thermoreceptor upstream of GPA-3 in ADL, the thermoreceptor and GPA-3 might change TRPV activity via a second messenger in ADL (Fig. 6).

The main molecular mechanisms underlying sensory signalling are evolutionally conserved from *C. elegans* to humans. Therefore, the molecular systems described in this study provide useful information for studying thermosensation in other organisms.

## Methods

### *C. elegans* strains

The wild-type N2 (Bristol) strain was used in all experiments. The following mutant strains were used: *osm-9(ky10)*, *ocr-2(ak47)*, *osm-9(ky10) ocr-2(ak47)*, *osm-9(ky10) ocr-2(ak47); ocr-1(ak46), osm-9(ky10);Ex[pAK62, pKDK66]*, *osm-9(ky10);Ex[srh-220p::osm-9cDNA, pAK62, pKDK66], ocr-2(ak47);Ex[pAK62, pKDK66], ocr-2(ak47);Ex[srh-220p::ocr-2cDNA, pAK62, pKDK66], ocr-2(ak47);Ex[sre-1p::yc3.60, pRF04]*, *ocr-2(ak47);Ex[srh-220p::ocr-2cDNA, sre-1p::yc3.60, pRF04], osm-9(ky10);Ex[sre-1p::yc3.60, pRF04]*, *osm-9(ky10);Ex[srh-220p::osm-9cDNA, sre-1p::yc3.60, pRF04], osm-9(ky10) ocr-2(ak47);Ex[sre-1p::yc3.60, pRF04], osm-9(ky10) ocr-2(ak47);ocr-1(ak46)Ex[sre-1p::yc3.60, pRF04], glr-3(tm6403);Ex[flp-6p::Ce-GCaMP8, gcy-5p::tagRFP]*, *glr-3(tm6403);Ex[flp-6p::Ce-GCaMP8, gcy-5p::tagRFP, gcy-5p::osm-9cDNA, gcy-5p::ocr-2cDNA]*, *glr-3(tm6403);Ex[flp-6p::Ce-GCaMP8, gcy-5p::tagRFP, gcy-5p::osm-9cDNA].* The original *osm-9(ky10)* and *ocr-2(ak47)* strains were previously outcrossed, and we used the same backcrossed strains reported in previous papers for this study^12,15^.

### Statistical analysis

Error bars in figures indicate standard errors of the mean (SEM). Statistical analyses were performed using ANOVA followed by Dunnett’s post-hoc test for multiple comparisons in Figs. 2a–c, 3a and 4, or by a Bonferroni multi-comparison test for results in Figs. 2d,e and 3b,c. Single (*) and double asterisks (**) indicate *p* < 0.05 and *p* < 0.01, respectively. For two-electrode voltage clamp in *Xenopus* oocytes in Fig. 5b,d, statistical analyses were performed using ANOVA followed by a Bonferroni multi-comparison test for results detected between groups marked with “a” and “b” (*p* < 0.05). See Supplemental Dataset S1 for further details on raw data and statistical figures.

### Temperature acclimatisation assay

A temperature acclimatisation assay was performed as previously described^5,10,15,51^. We used a 15 °C → 25 °C → 2 °C protocol. We used well-fed adult animals as they prepared to lay eggs. One animal was placed on a 3.5-cm plate of nematode growth medium (NGM) with 2% (w/v) agar and *E. coli* OP50. The adult animal was removed the following day and its progeny were cultured for 144–150 h at 15 °C. Approximately 100 animals on a plate were transferred to a 2 °C fridge after being at the optimal condition of 25 °C for 0, 3, or 5 h. After 48 h, plates were transferred to 15 °C and stored overnight. Numbers of dead and alive animals were recorded. Mutants were compared with wild-type animals for each temperature acclimatisation condition. When we carried out the analysis for Fig. 2b in winter to spring seasons, the survival rate of all animal strains were wholly increased compared with the results shown in Fig. 2d,e, which were carried out in the rainy season; thus, this observed difference may have been caused by humidity and other unknown factors, as mentioned in previous reports detailing the protocol^14,51^.

### In vivo Ca^2+^ imaging

In vivo Ca^2+^ imaging was performed essentially according to previous studies^5,12,52^. Yellow cameleon 3.60 (YC3.60) driven by the *sre-1* promoter was used as a genetically encoded Ca^2+^ indicator for Ca^2+^ imaging of ADL neurons. *osm-9* and *ocr-2* single mutants, the *osm-9 ocr-2* double mutant, and *osm-9 ocr-2; ocr-1* triple mutant expressing YC3.60 in ADL were constructed as previously described^15^*.* A *C. elegans* codon-optimised G-CaMP8 driven by the *flp-6* promoter was used as a genetically encoded Ca^2+^ indicator for Ca^2+^ imaging of ASER neuron^13^. pMIU34 *flp-6p::CeG-CaMP8* and pKOB006 *gcy-5p::tagRFP* was expressed in a *glr-3* mutant lacking the cold-sensitive kainate-type glutamate receptor in ASER*.* Animals were attached to a 2% (w/v) agar pad on glass, immersed in M9 buffer, coverslipped, and placed on an ITO glass-based thermocontroller (Tokai Hit Co., Fujinomiya, Japan) mounted on the stage of an Olympus IX81 or BX61 microscope (Olympus Corporation, Tokyo, Japan) for Figs. 3a–c and 4, respectively. Fluorescence was observed using a Dual-View (Molecular Devices, San Jose, CA) or W-View (Hamamatsu Photonics, Hamamatsu, Japan) optical system for Fig. 3a, and a split-view model of CSU-W1 (Yokogawa Electric Corporation, Tokyo, Japan) optical system for Figs. 3b,c and 4. YC3.60 donor and acceptor fluorescence signals, or G-CaMP8 and tagRFP fluorescence signals, were simultaneously captured using an EM-CCD camera with 1 × 1 binning, EVOLVE512 (Teledyne Photometrics, Tucson, AZ) for Fig. 3, and iXon Ultra 888 (Oxford Instruments, Abingdon, UK) for Figs. 3b, c and 4. Images were taken with 15-ms and 250-ms exposure times for Figs. 3a–c and 4, respectively. For each imaging experiment, fluorescence intensity was measured using the MetaMorph (Molecular Devices) image analysis system. Relative changes in intracellular Ca^2+^ concentrations were measured as changes in the YFP/CFP fluorescence ratio of YC3.60, or the green/red fluorescence ratio of G-CaMP8 and tagRFP. We used different Ca^2+^ indicators for each experiment depending on the result of previous studies. Previously, YC3.60 was used for Ca^2+^ imaging of thermosensory neurons regulating cold tolerance, such as ADL^5,12,15^. GCaMP8.0 was used for ectopic expression analysis of ASER in Takagaki et al.^13^ because the Ca^2+^ sensitivity of GCaMP8.0 is higher than YC3.60. When we carried out the analyses for Fig. 3b,c in rainy season, the ADL thermal responses of all animal strains were wholly decreased compared with results shown in Fig. 3a, which were carried out in winter to spring seasons. This result may be similar to the cold tolerance phenotype in that it may have been affected by humidity and other unknown factors, as previously mentioned in reports detailing the protocol^14,51^.

### Molecular biology

*osm-9* cDNA was amplified by PCR from an extrachromosomal array in the *C. elegans* transgenic strain SH231 *osm-9(ky10);pdrEx30[sre-1p::osm-9cDNA::gfp, unc-122::DsRed]*. *ocr-2* cDNA was amplified from an *ocr-2* cDNA in pCDNA3.1( +) gifted by Cori Bargmann (Rockefeller University). *ocr-1* cDNA was gifted by Cori Bargmann. pOX(+) contains *Xenopus* beta globin 5′- and 3′-untranslated regions. *osm-9*, *ocr-2*, and *ocr-1* cDNA were cloned into pOX(+) to synthesise cRNA for electrophysiological experiments in *Xenopus* oocytes [*osm-9* cDNA in pOX(+): pKOH220, *ocr-2* cDNA in pOX(+): pKOH226, *ocr-1* cDNA in pOX(+): pMIU084]. PCR fragments of *osm-9* and *ocr-2* cDNA from pKOH220 or pKOH226 were created by replacing the *CeG-CaMP8* of pMIU036 with *osm-9* or *ocr-2* cDNA (*gcy-5p::osm-9 cDNA*: pMIU091, *gcy-5p::ocr-2cDNA*: pMIU092). pMIU115 *srh-220p::osm-9cDNA* and pMIU116 *srh-220p::ocr-2cDNA* contain about 2500 bp upstream promoter sequence for the *srh-220* gene, respectively.

### Two-electrode voltage clamp in *Xenopus* oocytes

In vivo two-electrode voltage clamp in *Xenopus* oocytes was performed essentially according to previous studies^14,34^. Mature *X. laevis* females purchased from Hamamatsu Seibutsu Kyozai (Hamamatsu, Japan) were kept at 18–20 °C for oocyte collection. pOX(+) vectors, containing OSM-9, OCR-2, and OCR-1 were linearised with *MluI*, and complementary RNA (cRNA) was synthesised using the mMESSAGE MACHINE SP6 kit (Ambion, Austin, TX). OSM-9, OCR-2, and OCR-1 were singly expressed or co-expressed in *Xenopus* oocytes, and ionic currents were recorded using a two‐electrode voltage‐clamp method. Oocytes were collected from mature females and treated with collagenase A (Roche, Basel, Switzerland) to enzymatically remove follicular membranes. cRNA (50 nL) was injected into oocytes. The concentration of *osm-9*, *ocr-2,* or *ocr-1* cRNA injected into oocytes was 123 ng/μL, 118 ng/μL, or 111 ng/μL, respectively. Current recordings were performed at 5 or 6 days post-injection. Ionic currents were recorded using an OC-725C amplifier (Warner Instruments, Hamden, CT) with a 1-kHz low‐pass filter and digitised at 5 kHz by Digidata 1440 (Axon Instruments, Molecular Devices). Oocytes were voltage‐clamped at − 60 mV. Recording was performed at room temperature and warm-stimulation (~ 36 °C) was applied by perfusion of heated ND96 bath solution (in mM: 96 NaCl, 2 KCl, 1.8 CaCl_2_, 1 MgCl_2_, and 5 HEPES, pH 7.6). The temperature of perfused bath solutions was monitored with a TC-344B temperature controller (Warner Instruments) located just beside the oocytes. To obtained I–V relationships shown in Fig. 5e, ramp pulses were applied from − 80 to + 80 mV during 0.5-s at 3-s intervals.

### Ethical issues and approval

All animal treatments in this research were performed in accordance with the Japanese Act on Welfare and Management of Animals (Act No. 105 of October 1, 1973; latest revisions Act No. 51 of June 2, 2017, Effective June 1, 2018). All experimental protocols were approved by the Institutional Animal Care and Use Committees of Konan University and National Institute for Physiological Science.

## Supplementary information

Supplementary Information.

## Data Availability

The datasets generated during in this study are available from the corresponding author on reasonable request.
